# Association between tumor necrosis factor alpha-238G/a polymorphism and tuberculosis susceptibility: a meta-analysis study

**DOI:** 10.1186/1471-2334-12-328

**Published:** 2012-11-28

**Authors:** Zhijiao Zhang, Hang Zhu, Xudi Pu, Siying Meng, Fan Zhang, Lei Xun, Qin Liu, Yang Wang

**Affiliations:** 1 School of Public Health and Health Management, Chongqing Medical University, Chongqing 400016, China; 2 China Network of Effective Health Care Research Consortium, Chongqing Medical University, Chongqing 400016, China; 3 School of Foreign Language, Chongqing Medical University, Chongqing 400016, China; 4 School of Public Health, Chongqing Medical University, No 1 Yixueyuan Road, Chongqing 400016, P.R. China

**Keywords:** TNF-α, Polymorphism, Tuberculosis, Susceptibility, Meta-analysis

## Abstract

**Background:**

Tumor necrosis factor alpha (TNF-α) plays a key role in the containment of tuberculosis. The relationship between the TNF -238G/A polymorphism and tuberculosis susceptibility remains inconclusive. A comprehensive meta-analysis was made to provide a more precise estimate of the relationship between them.

**Methods:**

Multiple search strategies were used. A fixed effect model was taken*took* to estimate pooled OR with 95% confidence interval (CI) for the association between the TNF -238G/A polymorphism and tuberculosis susceptibility. The Chi-squared-based Q-test and I-squared*I*^*2*^ statistic were calculated to examine heterogeneity. Begg’s funnel plot and Egger’s test were used to assess publication bias.

**Results:**

9 case-control studies were included in this meta-analysis. No significant heterogeneity was demonstrated, and no obvious publication bias was detected among the included studies. The meta-analysis indicated that there was no significant association between the TNF -238G/A polymorphism and tuberculosis susceptibility (GA+AA versus GG model: OR=1.005, 95% CI: 0.765-1.319; A versus G model: OR=1.000, 95% CI: 0.769-1.300). In the subgroup analyses by ethnicity, types of TB and human immunodeficiency virus (HIV) status, no significant association were identified.

**Conclusions:**

The meta-analysis involving 2723 subjects did not detect any association between the TNF -238G/A polymorphism and tuberculosis susceptibility.

## Background

Tuberculosis (TB) is still a high-burden disease in present society. In spite of the preventability and curability of TB, there still remained 8.8 million incident cases, 12.0 million prevalent cases, and 1.4 million deaths in 2010 on a global scale [1]. Among those who are infected (approximately one third of the whole population), only an estimated 10% will develop clinical signs of disease while the majority of individuals are naturally resistant to TB [2]. Apart from environmental factors, genetic variability is regarded to be responsible for the advancement of infection [3]. Some gene polymorphisms have been demonstrated to be associated with TB susceptibility [4-6]. These significant findings may lead to the development of new TB preventive or therapeutic strategies [7].

Tumor necrosis factor alpha (TNF-α) is an immunoregulatory cytokine, which is produced primarily by monocytes and macrophages [8]. It plays an essential role in the proper regulation of host defense to tuberculosis in both animal models and human models [9,10]. The TNF-α gene cluster, which encodes the cytokine TNF-α, is located within the major histocompatibility complex (MHC) between human leukocyte antigen-B (HLA-B) and the HLA class III genes [11]. The single nucleotide polymorphisms (SNPs) as the most common genetic variation of TNF-α was considered to substantially influence the production capacity of TNF-α [12]. A few of them have been identified at positions−238, −308, −857, −863, and −1031 [13,14]. One of the extensively studied polymorphisms was at -238G/A position [15].

So far, there have been an increasing trend of researches conducted to assess the relationship between TNF-238 polymorphism and TB susceptibility, but the results remain inconclusive. Some studies have identified the significant association between the TNF-238G/A polymorphism and TB susceptibility [16,17]. Meanwhile, a contrary result emerged in a number of studies with TNF-238G/A polymorphism being not associated with TB [13,18-21]. Thus, we carried out a meta-analysis in order to estimate the association between the TNF-238G/A polymorphism and susceptibility to TB.

## Methods

### Search strategy

A literature search was conducted among six English databases (PubMed, Embase, Web of Science, Science Direct, SpringerLink and EBSCO) and two Chinese databases (Wanfang and Chinese National Knowledge Infrastructure databases) to identify studies involving association between tuberculosis susceptibility and TNF-238G/A polymorphism through October 2011. The search terms used (both in English and Chinese databases) were as follows: tuberculosis or TB or mycobacteria, polymorphism or variant or mutation or susceptibility, “tumor necrosis factor” or TNF or cytokine or gene.

### Study selection

Studies were included in the analysis if (1) evaluated the association between TNF -238G/A polymorphism and TB susceptibility, (2) was a case-control study, (3) it provided genotype frequencies or numbers for cases and controls to calculate odds ratios (OR), and (4) genotype distributions in control group must be consistent with Hardy–Weinberg equilibrium (HWE; p<0.05). We excluded studies if (1) the studies were reviews or abstracts, (2) Its targets were animals rather than human beings, (3) genotype frequencies or numbers were not reported, (4) studies were conducted among the patients with some potential confounding diseases, such as pneumoconiosis. For overlapping and republished studies, only the most recent or complete study was included. Two of the researchers independently reviewed the full text of all studies to ensure that the studies could meet preset criteria for the inclusion. Any discordance between the two researchers was subsequently resolved through discussion.

### Data extraction

Two reviewers independently collected the data, cross-checked and reached a consensus on all items. The following items were extracted from each study: First author, publication year, original country, ethnicity, sample size, diagnostic methods of cases, selection criteria of controls, genotype and allele number in cases and controls.

### Statistical analysis

The strength of association between TNF -238G/A polymorphism and TB susceptibility was estimated by OR with the corresponding 95% confidence interval (CI). The genetic model evaluated for pooled OR was as follows: AA + AG versus GG and A versus G. The significance of the pooled OR was determined by Z test. Heterogeneity among studies was checked by a χ^2^-based Q test and I^2^ statistic [22,23]. When the heterogeneity was not considered significant, the pooled OR was calculated by the fixed-effects model (Mantel–Haenszel) [24,25]. Otherwise a random-effects model was used (DerSimonian and Laird) [25]. Subgroup analysis was performed by ethnicity, HIV status and TB type to produce accurate results. Sensitivity analysis was conducted by omitting one single study each time, and leaving out studies specifically on smear-confirmed TB, and restricting the analysis specifically on culture-confirmed TB. Publication bias was examined visually in a Begg’s funnel plot and Egger’s test [26,27]. HWE was tested by Fisher’s exact test via Genepop on web [28,29], and other analyses were performed using STATA version 12.0 software (Stata Corporation, College Station, TX, USA). All tests were two-sided and the significance level*s* was 0.05.

## Results

### Selection process and characteristics of eligible studies

**Figure 1:**
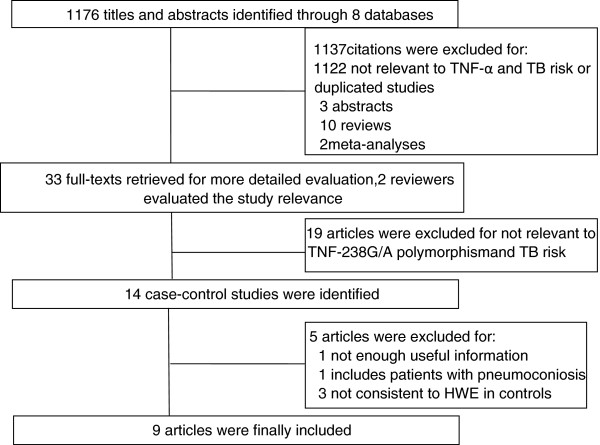
Flow diagram of selection process.

A total of 1172 titles and abstracts were found from eight selected electronic databases mentioned above. As shown in Figure 1, 33 potential studies were retrieved for a detailed full-text evaluation. 19 studies talking about the association between other polymorphisns of TNF-α and TB were excluded. Therefore, 14 studies were left for further selection. One study [30] with inadequate genotype or relevant information, one study [31] targets TB patients with complications of other diseases (pneumoconiosis patients) and another three studies [16,17,32] inconsistent with HWE in controls were excluded. Finally, nine case-control studies [13,14,18-21,33-35] in total were included in the meta-analysis.

Among nine selected studies, which were all published from 2001 to 2011, eight studies were written in English and one study [35] in Chinese, while six were conducted in Asian populations, two in Caucasians and one in Africans (Table 1). A total of 2723 participants (1184 cases and 1539 controls) were analyzed, with the sample sizes ranging from 86 (46 cases and 40 controls) to 714 (181 cases and 533 controls). Most cases were diagnosed by acid-fast bacilli on sputum smear or confirmed by tuberculosis on sputum culture. Eight studies described specific types of TB, consisting of five PTB studies, one EPTB study, and two studies focused on both PTB and EPTB. HIV status of the cases and controls was supplied in three studies, and only HIV-negative TB cases were included. All the controls were health irrelevant individuals, and seven of which were well matched. Genotype and allele distributions for each study were presented (Table 2). Hardy-Weinberg equilibrium in controls was tested by Fisher’s exact test via Genepop on web and the results were shown below.

**Table 1:** Characteristics of included studies

**Author**	**Country (ethnicity)**	**Cases/controls**	**Diagnosis of cases**	**Selection of controls**
Selvaraj	India (Asian)	210/120	Smear, culture, radiological, clinical response confirmed PTB	Normal healthy spouses and Tuberculosis Research Centre staff, ethnicity and area of residence matched
Fitness	Malawi (African)	181/533	Smear, culture, histology confirmed TB, HIV negative	No history or symptom of TB; not first or second degree relatives of the cases; age, sex and area of residence matched
Vejbaesya	Thailand (Asian)	149/147	Smear, culture, clinical and radiological response confirmed PTB, HIV negative	Unrelated healthy blood bank donors
Ates	Turkey (Caucasian)	128/80	Smear, culture, pathological, radiological , clinical response confirmed PTB,EPTB	No history of TB noted on chest radiography, age and sex matched
Trajkov	Republic of Macedonia (Caucasian)	75/301	Smear/culture confirmed PTB	No family history of TB, nationality and area of residence matched
Merza	Iran (Asian)	117/60	Smear and radiological confirmed PTB	Nurses, doctors and TB staff with no laboratory or clinical sign of diseases development, age, sex and nationality matched
Sharma	India (Asian)	185/155	Smear, culture, histology, radiological , clinical response confirmed PTB,EPTB,HIV negative without chronic illness	Without a family history of TB or any other related disease and a possibility of a latent tuberculosis infection, socioeconomic status and ethnicity matched
Anoosheh	Iran (Asian)	93/103	Smear and culture confirmed PTB	No history of TB
Lin	China (Asian)	46/40	Pathological examination and clinical response confirmed EPTB	NR

*NR=not reported; HIV = human immunodeficiency virus; PTB= pulmonary tuberculosis; TB = tuberculosis; EPTB= extrapulmonary tuberculosis; HWE= Hardy–Weinberg equilibrium.

**Table 2:** Genotype and allele distributions in cases and controls

**First author**	**Cases**					**Controls**					**HWE**
	**GG**	**GA**	**AA**	**G**	**A**	**GG**	**GA**	**AA**	**G**	**A**	
Selvaraj	176	34	0	386	34	96	23	1	213	25	1
Fitness	162	19	0	343	19	464	69	0	997	69	0.1536
Vejbaesya	136	13	0	285	13	137	10	0	284	10	1
Ates	121	7	0	249	7	77	3	0	157	3	1
Trajkov	70	5	0	145	5	276	23	2	575	27	0.1089
Merza	108	9	0	225	9	57	3	0	117	3	1
Sharma	164	19	2	347	23	141	14	0	296	14	1
Anoosheh	84	9	0	177	9	98	5	0	201	5	1
Lin	43	3	0	89	3	37	3	0	77	3	1

*HWE= Hardy–Weinberg equilibrium.

### Quantitative synthesis

**Figure 2:**
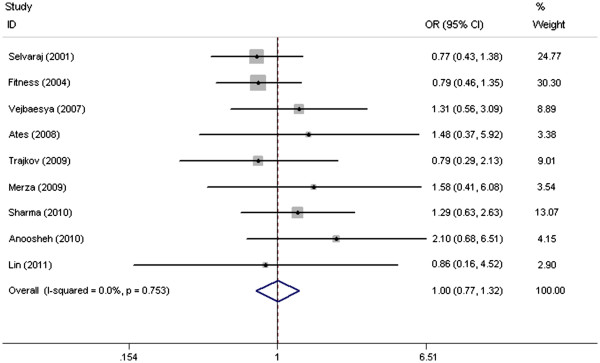
Forest plots of associations between TNF-238 SNP and TB susceptibility in GA+AA vs GG model.

**Figure 3:**
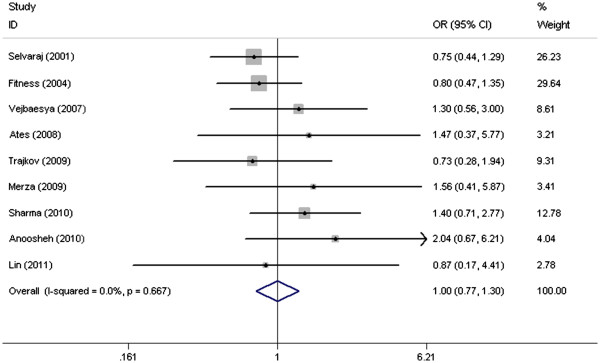
Forest plots of associations between TNF-238 SNP and TB susceptibility in A vs G model.

Based on the results in the meta-analysis, no significant heterogeneity was observed between the eligible studies, both in GA+AA versus GG genotype model and A versus G genotype model, with P-value for χ^2^ being 0.753 and 0.667 respectively; I^2^, another index of heterogeneity test, was the same 0.0% in two genotype models. Therefore, we chose fixed-effects model to analyze the pooled OR, and found no significant association between the TNF-238G/A polymorphism and TB susceptibility (GA+AA versus GG model: OR=1.005, 95% CI=0.765–1.319, P=0.974; A versus G genotype model: OR=1.000, 95% CI=0.769–1.300, P=1.000) (Figure 2 and Figure 3).

We further tried to carry out the subgroup analyses on basis of different ethnicity, clinical types of TB and HIV status (Table 3). However, no material association was found.

**Table 3:** Pooled ORs of association between TNF-238G/A polymorphism and TB susceptibility

**Comparison**	**Cases/Controls**	**GA+AA versus GG**			**A versus G**		
		**OR(95% CI)**	**P**_**z test**_	**P**_**h**_	**OR(95% CI)**	**P**_**z test**_	**P**_**h**_
**Ethnicity**							
**Asian**	800/625	1.124 (0.792–1.597)	0.639	0.513	0.513 (0.799–1.567)	0.538	0.513
**Non-Asian**	384/914	0.844 (0.542-1.313)	0.451	0.698	0.837 (0.544-1.287)	0.417	0.687
**TB type**							
**PTB**	644/731	1.037 (0.708-1.517)	0.853	0.487	0.993 (0.689-1.431)	0.971	0.428
**Other TB**	359/275	1.259 (0.698-2.271)	0.443	0.878	1.334 (0.755-2.358)	0.321	0.856
**HIV status**							
**HIV-**	515/835	1.003 (0.688–1.462)	0.989	0.446	1.034 (0.719–1.487)	0.855	0.375
**ND**	669/704	1.007 (0.679-1.493)	0.973	0.634	0.964 (0.661-1.407)	0.850	0.584

*P_h_= P_heterogeneity_; HIV-= HIV negative; PTB= pulmonary tuberculosis; ND= Not done.

We excluded each case-control study step by step and removing the TB patient confirmed by smear to test the sensitivity of the meta-analysis, and the corresponding pooled ORs received were not substantially different. (data not shown).

**Figure 4:**
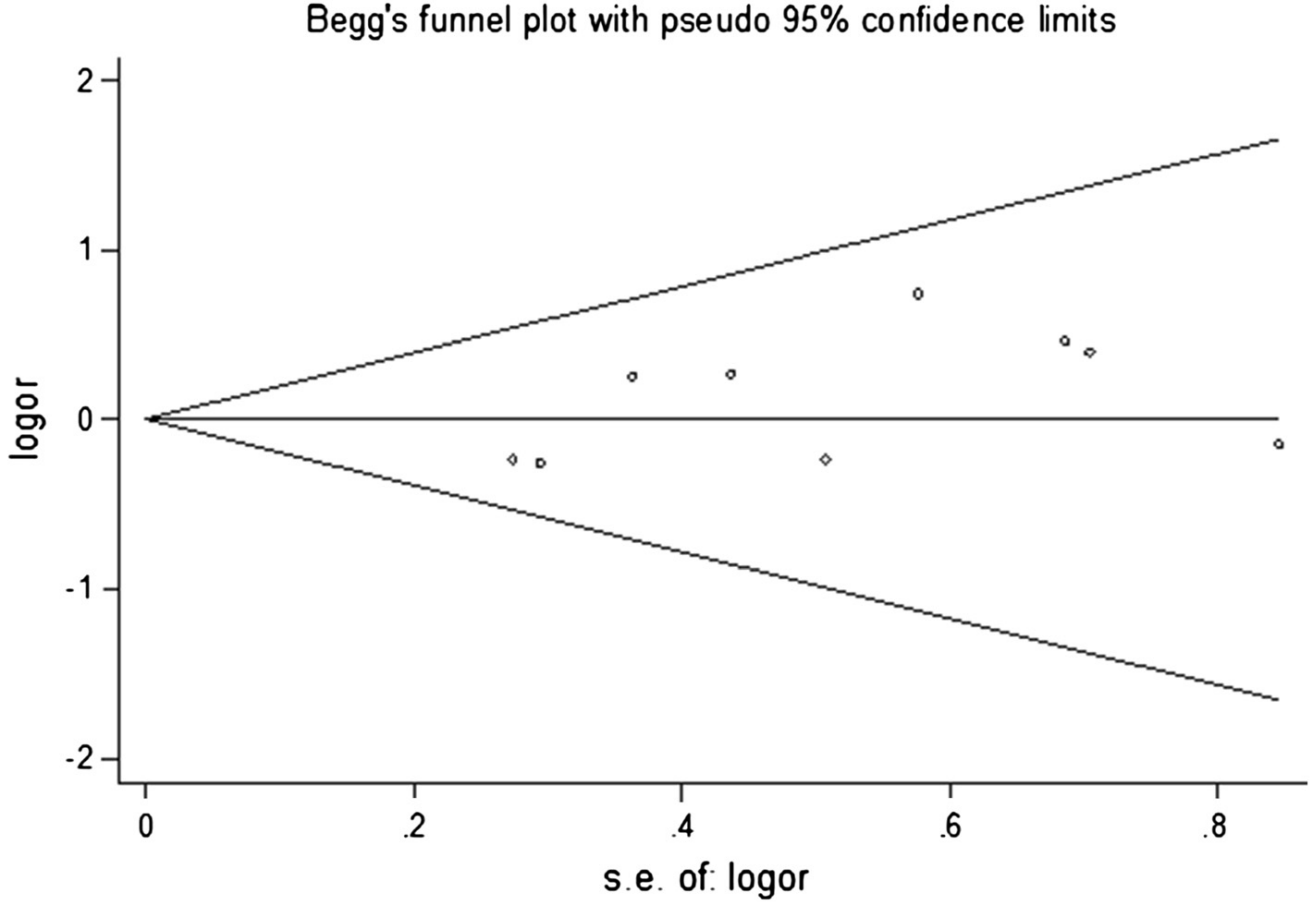
Begg’s funnel plot for publication bias in GA+AA vs AA genotype model.

**Figure 5:**
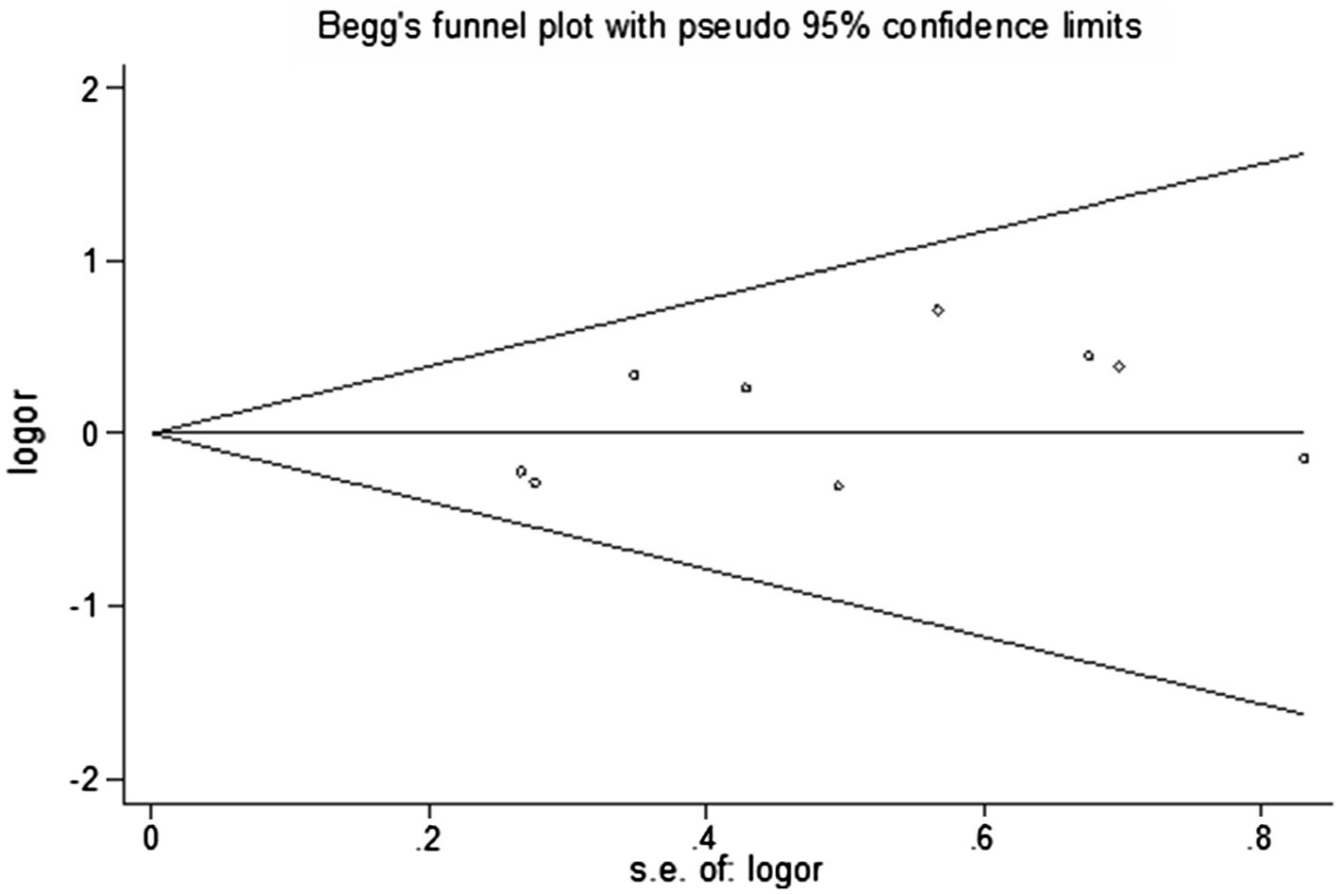
Begg’s funnel plot for publication bias in A vs G genotype model.

Publication bias among the eligible studies was assessed by the Begg’s funnel plot and Egger’s test (Figure 4 and Figure 5). No obvious asymmetry was revealed in either the GA+AA versus GG genotype model and A versus G genotype model. Therefore, Egger’s test was used to provide the evidence of publication bias, and no significant asymmetry was obtained (GA+AA versus GG model: P=0.094; A versus G model: P=0.130).

## Discussion

The hypothesis that TNF gene polymorphisms may increase the risk of developing TB has prompted a number of studies [7,36]. However, currently available published data on the association between the TNF-238G/A and the risk of TB provided contradictory results. On the one hand, positive associations might be spurious. On the other hand, the negative results might be a consequence of inadequate statistical power [37]. Therefore, this meta-analysis was conducted, and no association between the TNF-238G/A and TB susceptibility was identified.

The failure of this meta-analysis which did not detect any association between TNF -238 G/A polymorphism and TB susceptibility was attributed to several factors. First of all, other polymorphisms within the TNF gene impacting the production capacity of TNF-α may also affect the susceptibility to TB. The association between -308 polymorphism of TNF-α and TB susceptibility has been identified recently [38]. Moreover, it may be explained on the basis of linkage disequilibrium between other SNP that are directly linked to TB and TNF-α 238 site. The infrequent allele A of TNF-238 was in strong linkage disequilibrium with HLA-A1, and the latter one has been proved to be associated with bacteriological relapse [18]. In addtion, it may be on account of the opposing effect that exists between TNF and other genes. The balance between TNF-α and IL-10 was considerate to be important for control or dissemination of TB [39].

There are some limitations in this meta-analysis. Firstly, the cases were not uniformly defined. In some studies, cases were defined using smear microscopy and radiology while other studies used culture-confirmed TB as cases. Three of nine studies provided HIV status of cases, and it is better for the future researchers to mention their cases’ HIV status in the study since coinfections may influence the genetic susceptibility to TB. Secondly, a few studies described source of the controls, and it is important to note that population-based study may not have the same representativeness with hospital-based study. Thirdly, the subgroup analyses were only implemented among some explicitly described population due to the lack of original studies. Studies conduct among Non-Asian population, EPTB patients and HIV-positive TB patients were needed in the future.

Despite these limitations, there are some advantages in the meta-analysis. First, the HWE test was performed for the control groups in each study, and three studies that deviated from HWE were excluded in the process to ensure the homogeneity of the population genetic background. Second, Begg’s and Egger’s tests did not find any publication bias, indicating that the results were unbiased. Third, subgroup analyses and sensitivity analyses were conducted and the results were stable. As far as we were concerned, this is the first meta-analysis carried out so far to investigate the association of the TNF-238G/A polymorphism with TB.

## Conclusion

No apparent association between TNF-238G/A and TB susceptibility was identified in the meta-analysis. In the future, well-designed studies with large sample size among Non-Asian and EPTB population and with multiple SNPs are still needed due to the lack evidence in these fields.

## Competing interests

The authors declare that they have no competing interests.

## Authors’ contributions

ZZ, HZ and YW created the concept and design of this study. ZZ and XP conducted the literature search and extraction of data. XL and FZ were responsible for the statistical analysis. ZZ and HZ drafted the manuscript. QL and YW revised and edited the study. SW was in charge of English editing. All authors read and approved the final manuscript.

## Pre-publication history

The pre-publication history for this paper can be accessed here:

http://www.biomedcentral.com/1471-2334/12/328/prepub
